# Development of mixed starter culture for the fermentation of Ethiopian honey wine, *Tej*

**DOI:** 10.1038/s41598-022-17594-1

**Published:** 2022-08-04

**Authors:** Eskindir Getachew Fentie, Minsoo Jeong, Shimelis Admassu Emire, Hundessa Dessalegn Demsash, Min-Chul Kim, Kyeongmo Lim, Jae-Ho Shin

**Affiliations:** 1 College of Biological and Chemical Engineering, Addis Ababa Science and Technology University, 16417 Addis Ababa, Ethiopia; 2 School of Chemical and Bio-Engineering, Addis Ababa Institute of Technology, Addis Ababa University, King George VI Street, P.O. Box 385, 16417 Addis Ababa, Ethiopia; 3 Department of Applied Biosciences, Kyungpook National University, Daegu, 41566 Republic of Korea

**Keywords:** Applied microbiology, Industrial microbiology

## Abstract

Ethiopian honey wine is one of the country's most popular spontaneously fermented traditional alcoholic beverages. However, the final product of this natural fermentation system is frequently of poor and inconsistent quality. Furthermore, it makes the process difficult to predict, control, and correct. Thus, the main aim of this study was to develop a direct fermentation system for Ethiopian honey wine, *Tej*. After isolating fermentative microbial strains from *Tej* samples, they were subjected to intensive screening to fit to its purpose. Later, phenotypic and genotypic characterization, and inoculation of isolates to honey-must were performed sequentially. Finally, microbial interaction and physicochemical analysis, including volatile compounds profiling, were done for the inoculated samples. The identified isolates were strains of *Saccharomycetaceae* and *Lactobacillaceae* families. These strains showed a good ability to tolerate osmotic stress and a lower pH environment. *Tej* sample produced by mixed culture inoculation of *Saccharomyces* and *Lactobacillus* species showed similar physicochemical, volatile compounds, and sensory attributes values with that of the control sample. Thus, a mixture of *Saccharomyces* and *Lactobacillus* strains could be used as a starter culture to produce Ethiopian honey, *Tej,* without scarifying of its major quality attributes.

## Introduction

Traditional fermented foods and beverages are made from locally available raw materials using native microorganisms, indigenous knowledge and locally available utensils^1^. In Ethiopia, the production and consumption of traditional foods and beverages is a very common practice^2^. Ethiopian honey wine, *Tej*, is one of the popular traditional alcoholic beverages in the country^3^. It is typically a household commercial product sold for consumption at the point of production^3,4^. Like other traditional alcoholic beverages, *Tej* is produced by spontaneous fermentation of crude honey^5,6^. Due to the absence of direct inoculation, the fermentative microorganisms responsible for converting honey-water mixture to ethanol during *Tej* fermentation are thought to be found in raw materials and utensils^2^. This spontaneous honey fermentation could be the primary cause of inconsistency in the quality of *Tej* produced by different households^7,8^. The honey quality difference might also contribute to this variation in the physicochemical properties of the final product^6^.

Traditional *Tej* producers prefer crude honey instead of purified honey for the making a good quality *Tej*^5^. This crude honey is first mixed with water at a proportion of 1:3. Because of the increase in the price of honey, some producers substitute some portion of honey with cane sugar during *Tej* making^4^. The mixture is then left in mixing tank for an additional 3–4 days to complete its primary stage fermentation. It will then be filtered using clean cloth to remove sediment and suspended particles. Subsequently, leaves and stems of “gesho” (*Rhamnus prinoides*) are added to the previously fermented honey-water mixture. “gesho” (*R. prinoides*) is primarily added to mixture to enhance the flavor of final product^4^. Again, some *Tej* makers may also add barks from a selected tree and/or herbal ingredients to add a distinctive flavor to *Tej*^5^. The mixture is again allowed to ferment for an additional 8–21 days in anaerobic conditions to complete the final fermentation stage. After the mentioned fermentation period, it is now ready for consumption as a final product. Customers typically prefer *Tej*, which has a yellow color, a sweet taste, and a turbid appearance^6^.

So far, limited studies had been conducted towards to the development of direct inoculated *Tej* fermentation system. The very first full article on *Tej* by Bahiru et al.^6^ reported the physicochemical properties of *Tej* samples collected from Addis Ababa, Ethiopia. This report shows a considerable variation in some physicochemical properties for *Tej* samples collected from various households. After five years, the same authors reported the yeast and lactic acid flora of *Tej* by using phenotypic characterization method^4^. Similarly, *Lactobacillus* and *Saccharomyces* were the dominant taxa for the *Tej* samples collected from different part of the country^7^. However, the dominance level was substantially different within and across the samples collected from different households. Furthermore, as the fermentation period drew close to an end, the microbial dynamics moved to this microbial dominance^9^. Moreover, *Tej* exhibited good antioxidant activity, possibly inherited from honey and “gesho” (*R. prinoides*)^7^. The antioxidant capacity of *Tej* was also increased as the fermentation progressed to completion^9^. The ability of fermentative microorganisms to produce polyphenol and flavonoid through a complex microbial enzymatic system, as well as the continuous extraction of bioactive compounds from gesho as fermentation progressed to the end, are the most likely explanations for the increase in antioxidant activity during the *Tej* fermentation period^9–11^.

Previous reports on the physicochemical and microbial ecology of *Tej* did not provide enough information to modernize this spontaneous fermentation system^4,6–8^. The selection of starter culture strains and process variable optimizations are still open gaps to be filled for the complete transformation of this process. However, inoculated fermentation using single strain cultures might cause the fermented products to lose their peculiarity^12^. The main reason for this drawback is that the basis for selecting single strains is to minimize or maximize only one output function of the process^13^. Using a mixed culture is the best option for dealing with the drawbacks caused by both spontaneous and single strain inoculated fermentation systems^14^. Besides, using commercially available strains as a starter culture may result in substrate incompatibility, inability to adapt to stressful fermentation conditions, and/or loss of distinctive final product characteristics^13,14^. Thus, the purpose of this study was to isolate, screen, and characterize potential mixed starter cultures from *Tej* samples collected from various households, with the ultimate goal of developing an inoculated fermentation system for this traditional alcoholic beverage. Furthermore, we analyzed the physicochemical and volatile compounds to assess whether the isolated strain inoculations achieved the intended quality attributes of the final product.

## Results

### Isolation, characterization and screening of starter cultures

Based on macroscopic feature differences, 91 bacterial and 83 fungal colonies were isolated from *Tej* samples using de Man, Rogosa, and Sharpe (MRS) and yeast extract peptone dextrose (YPD) media. The catalase activity, gram reaction, and indole test to characterized the bacterial isolates. Indole negative, gram-negative, and catalase-negative characteristics were found in 67 of the 91 isolates. These bacterial and fungal isolates were subjected to further physiological tests. Isolates that could ferment carbohydrates and produce gas advanced to the next level of screening, which included the ability to grow at lower pH, varying temperatures, and higher sugar concentrations (Table 1). As a result, the number of isolates was reduced as the screening progressed to the end. A total of 34 presumptive heterofermentative *Lactobacillus* that could ferment carbohydrates and produce gas were screened from 67 bacterial isolates. Similarly, 49 fungal isolates that had a good ability to ferment carbohydrates were screened from the first isolated pool (Table 1). Subsequently, 27 and 31 bacterial and fungal isolates with a good ability to proliferate at lower pH (4.5) growth media were again screened. In this study, the isolates with an optical density (OD) value greater than 1.0 at 600 nm wavelength were considered as higher pH tolerant microbes. From the prior pool of isolates, 21 and 20 bacterial and yeast isolates were screened for their good ability to grow in extreme temperatures (Table 1). Finally, the ability of the isolates to grow at a higher sugar concentration was performed by using glucose concentrated MRS and YPD media. A total of 6 and 8 bacterial and yeast isolates with desirable characteristics were selected and subjected to genotypic identification and characterization.

**Table 1:** Physiological tests for purpose-oriented screening of bacterial and yeast isolates from *Tej* samples.

Physiological tests	Characteristic results	Number of isolates	Percent of isolates
Bacterial isolates		91	
Gram reaction	Negative	82	90.11%
Indole test	Negative	89	97.80%
Catalase activity	Negative	78	85.71%
**Carbohydrate fermentation test and gas production**			
Glucose	Positive	50	54.94%
Sucrose	Positive	41	45.05%
Ability to grow at a lower pH (4.5)	++	27	29.67%
**Ability to grow in different temperature**			
15 °C	++	25	27.47%
35 °C	+	23	25.27%
**Ability to grow at high sugar (glucose) concentration**			
10%	++	20	21.97%
15%	++	14	15.38%
20%	+	11	12.09%
Fungal isolates		83	
**Carbohydrate fermentation test**			
Glucose	Positive	68	81.92%
Sucrose	Positive	62	74.69%
Ability to grow at a lower pH (4.5)	++	31	37.35%
**Ability to grow in different temperature**			
15 °C	++	28	33.73%
35 °C	+	24	26.37%
**Ability to grow at high sugar (glucose) concentration**			
10%	++	19	22.89%
20%	++	13	15.66%
30%	+	10	12.05%

+Isolates that had recorded 0.8–1.0 OD values at 600 nm after 24 h incubation period.

++Isolates that had recorded greater than 1.0 OD values at 600 nm after 24 h incubation period.

### Molecular identification of isolates

**Figure 1:**
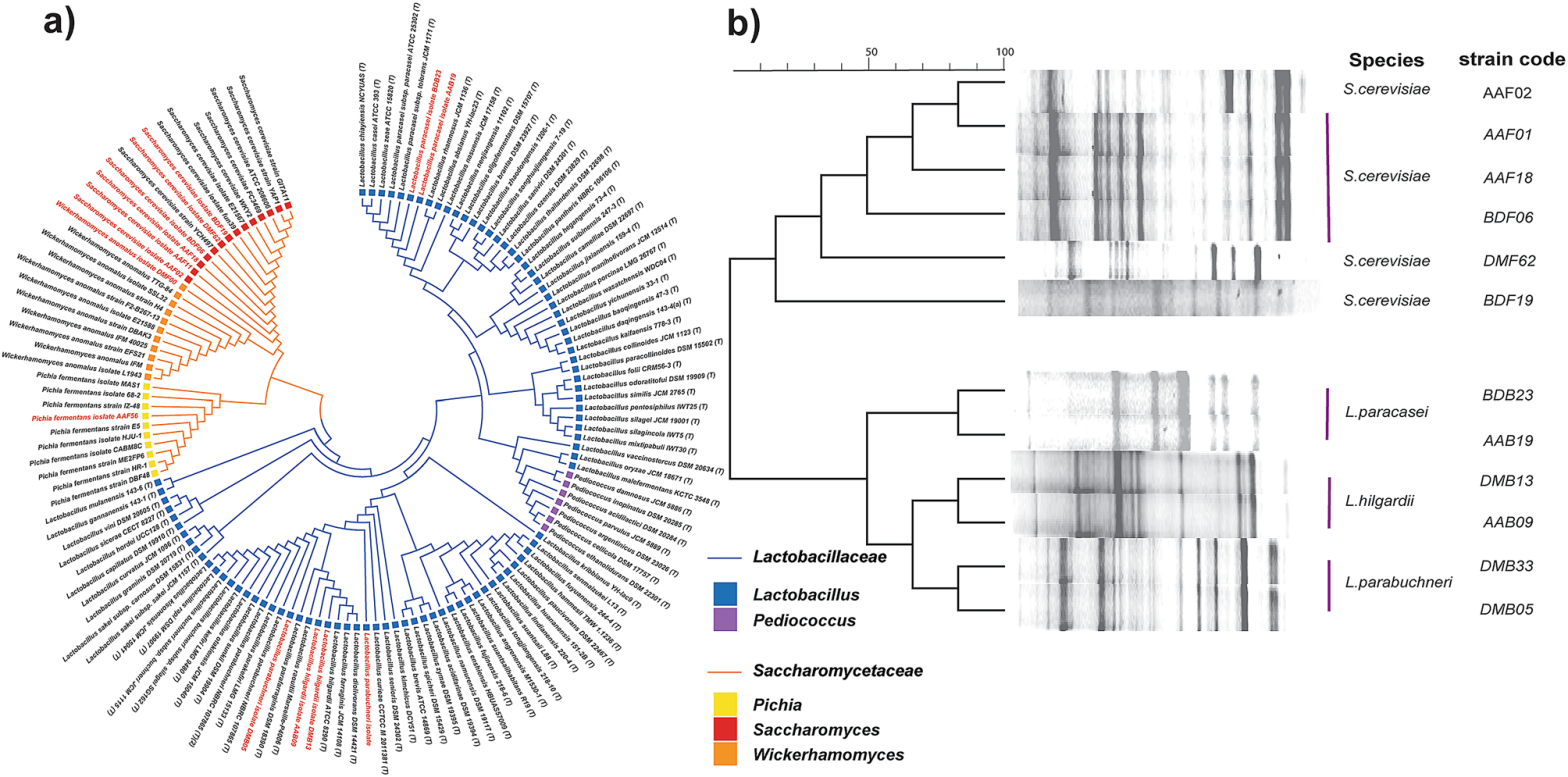
Phylogenetic tree of (**a**) *Lactobacillaceae* and *Saccharomycetaceae* isolate family members together with related species based on 16SrRNA and ITS gene sequencing data (**b**) Simple sequence repeats (SSR) data for 12 isolates of *Lactobacillaceae* and *Saccharomycetaceae*

Genotypic identification of bacterial and yeast isolates was performed using 16SrRNA and ITS amplicon sequencing. The morphological features and genotypic identification of the isolates is presented in Table 2. In addition, the phylogenetic tree based on the 16S rRNA gene and ITS amplicon sequenced data of the isolates together with the closely related species is illustrated in Fig. 1a. All bacterial isolates had shown greater than 98.5% similarity with the National Center for Biotechnology Information (NCBI) nucleotide sequence database (Table 2). Moreover, all isolated bacteria were a family of *Lactobacillaceae* (Fig. 1a). *Lactobacillus hilgardii*, *Lactobacillus paracasei,* and *Lactobacillus parabuchneri* were the identified species from 6 bacterial isolates (Table 2). The source of these isolates was *Tej* samples collected from different locations (Table 2). For instance, *L. parabuchneri* strains were isolated from *Tej* samples collected from Debre Markos, Ethiopia. Similarly, the rest two of *Lactobacillaceae* isolates were identified as *L. paracasei*. These strains were isolated from *Tej* samples collected from Addis Ababa and Bahir Dar, Ethiopia (Table 2). Based on ITS sequence data, identified isolates were the species of *Saccharomycetaceae* (Fig. 1a). *Saccharomyces cerevisiae*, *Pichia fermentans*, and *Wickerhamomyces anomalus* were the identified species from the collected *Tej* samples (Table 2). Six out of eight isolates were identified as *S. cerevisiae* strains. The strains of this species were isolated from *Tej* samples collected from all source locations. The other identified isolate using ITS sequence was *P*. *fermentans* strain. This strain was isolated from *Tej* samples collected from Bahir Dar and Addis Ababa, Ethiopia. The remaining isolate, *W. anomalus* strain, was isolated from *Tej* samples collected from Debre Markos (Table 2). Furthermore, phylogenetic relationship and genetic diversity analysis were conducted for the isolated strains of the same species. Randomly amplified polymorphic DNA polymerase chain reaction (RAPD-PCR) and Microsatellite multiplex PCR were used to achieve these objectives. All *S. cerevisiae* isolates were subjected to the above-mentioned analysis, with the exception of *P. fermentans* and *W. anomalus*, which were isolated only once. All strains of *Lactobacillus* species had clustered very narrowly together with very uniform DNA fragment pattern (Fig. 1b). Whereas, the strains of *S. cerevisiae* showed a higher level of polymorphism (Fig. 1b). Three strains of *S. cerevisiae* had shown a uniform DNA fragment pattern. The rest three *S. cerevisiae* strain isolates had shown a difference in the pattern of amplified DNA fragment on agarose gel electrophoresis. *S. cerevisiae* with the strain code AAF18 was chosen for further analysis and inoculation honey-must in this study based on the majority in similarity of presence. Since strains of the same species of *Lactobacillus* showed high level of genome similarity, we used a strain code DMB13, DMB33, and DMB23 to be used as mixed culture inoculum for honey-must fermentation.

**Table 2:** Morphological characteristics and genotypic identification of bacterial and fungal isolates.

Isolate strain code	Source	Morphological characterization						Genotypic identification		GenBank accession number
		Shape	Size	Pigment	Surface	Elevation	Opacity	Species	Percent Identity	
**Bacterial isolates**										
DMB13	Debre Markose	Round	Medium	White	Glistening	Flat	Opaque	*Lactobacillus hilgardii*	99.80	AB911494.1
DMB33	Debre Markose	Round	Medium	White	Smooth	Flat	Opaque	*Lactobacillus parabuchneri*	99.61	CP018796.1
AAB09	Addis Ababa	Punctiform	Small	Creamy	Smooth	Pulvinate	Opaque	*Lactobacillus hilgardii*	99.67	AB911494.1
BDB23	Bahir Dar	Punctiform	Small	Creamy	Glistening	Flat	Opaque	*Lactobacillus paracasei*	99.93	KR816165.1
DMB05	Debre Markose	Irregular	Medium	Creamy	Glistening	Convex	Translucent	*Lactobacillus parabuchneri*	99.41	FJ476125.1
AAB19	Addis Ababa	Punctiform	Small	White	Smooth	Flat	Opaque	*Lactobacillus paracasei*	99.60	KR816165.1
**Yeast isolates**										
AAF02	Addis Ababa	Round	Large	White	Smooth	Umbonate	Opaque	*Saccharomyces cerevisiae*	99.61	MK267684.1
AAF11	Addis Ababa	Round	Large	White	Glistening	Umbonate	Opaque	*Saccharomyces cerevisiae*	98.97	CP006424.1
AAF18	Addis Ababa	Round	Medium	Creamy	Smooth	Raise	Translucent	*Saccharomyces cerevisiae*	99.48	MK267684.1
DMF00	Debre Markose	Round	Medium	Creamy	Smooth	Flat	Opaque	*Wickerhamomyces anomalus*	99.46	MT321267.1
BDF06	Bahir Dar	Round	Large	White	Smooth	Pulvinate	Opaque	*Saccharomyces cerevisiae*	99.35	MK267684.1
AAF56	Addis Ababa	Round	Medium	White	Dull	Convex	Opaque	*Pichia fermentans*	97.14	MT645425.1
DMF62	Debre Markose	Round	Large	Creamy	Glistening	Flat	Opaque	*Saccharomyces cerevisiae*	99.61	LC215450.1
BDF19	Bahir Dar	Round	Large	White	Smooth	Umbonate	Translucent	*Saccharomyces cerevisiae*	98.97	CP006424.1

### Phenotypic properties of isolates

**Figure 2:**
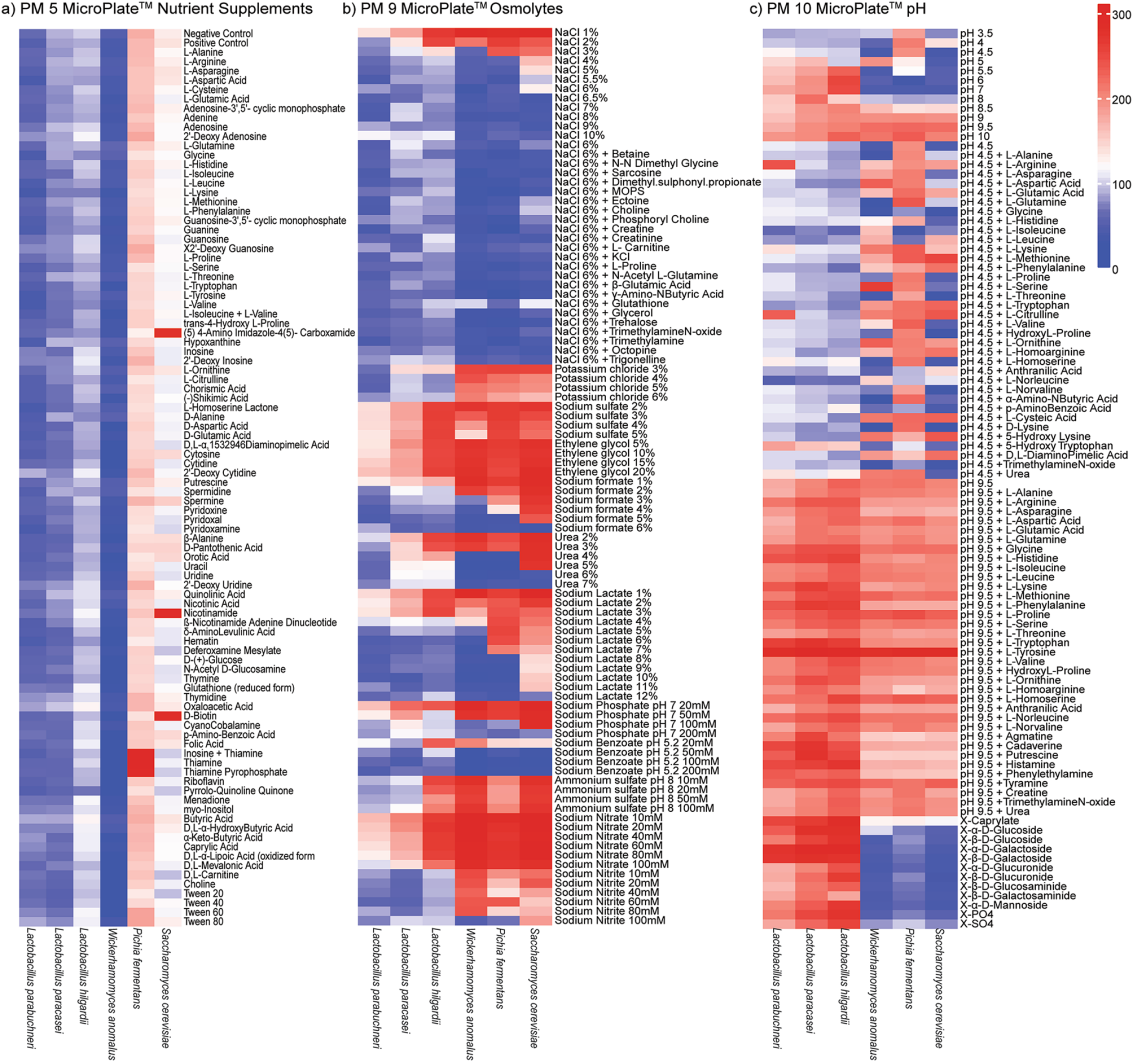
Heat map of the phenotypic microarray results for *Saccharomycetaceae* and *Lactobacillaceae* isolates using different (**a**) Nutrient supplements (**b**) Osmolytes (**c**) pH microplates. The magnitude of microbial metabolism is illustrated by a different heatmap color. The larger magnitude is indicated by dark red, while the magnitude gradually falls to light red, white, light blue, and finally dark blue, with the latter denoting a lesser magnitude.

The characterization of isolates was further strengthened by phenotypic microarray (PM) analysis. PM 5, PM 9, and PM 10 microplates were used to achieve this purpose. The nutritional requirement of the isolates was determined using PM 5 microplate. PM 9 and PM 10 were utilized to evaluate the reaction of the isolates to osmotic stress and extreme pH environment. The isolated *S. cerevisiae* showed the best metabolism at the medium supplemented with D-biotin, nicotinamide, and (5)4-amino imidazole-4(5)-carboxamide (Fig. 2a). While the metabolism of *P. fermentans* isolate was best at the medium supplemented with inosine + thiamine, thiamine, and pyrophosphate. However, *W. anomalus* and almost all *Lactobacillus* isolates did not have any special preference over the nutrient supplements available on PM 5 (Fig. 2a). All *Saccharomycetaceae* isolates recorded a good metabolism at different concentrations of sodium nitrite, sodium lactate, sodium formate, sodium sulfate, ethylene glycol, and potassium chloride. Nevertheless, lower metabolism was recorded for these isolates in the growth medium containing high sodium chloride (NaCl) concentration (Fig. 2b). Similarly, all *Lactobacillus* isolates responded well to increased sodium nitrite, sodium sulfate, and ethylene glycol concentrations. Like *Saccharomycetaceae* isolates, all the *Lactobacillus* isolates had difficulties for proper metabolism at a higher NaCl concentration. (Fig. 2b). Furthermore, PM 10 characterization revealed that *Saccharomycetaceae* isolates were more adaptable to lower pH environments than *Lactobacillus* species. The isolates of *Lactobacillus* species responded well to medium having pH values greater than or equal to 4.5 (Fig. 2c). Compared to other *Saccharomycetaceae* isolates, *P. fermentans* responded relatively better to different pH environments. Although they are not the same as *P. fermentans*, *S. cerevisiae* and *W. anomalus* had also demonstrated acceptable tolerance to a different pH condition (Fig. 2c).

### Interaction between dominate species

**Figure 3:**
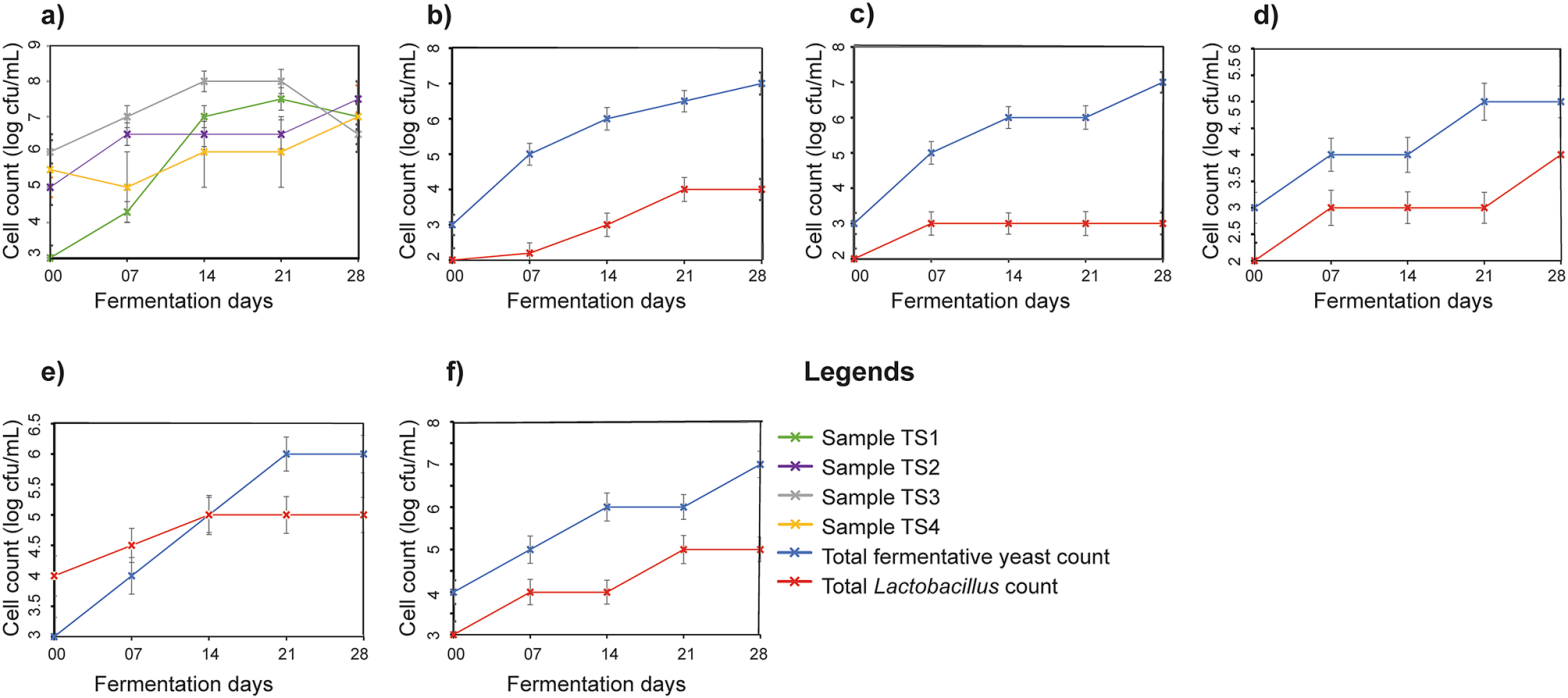
Microbial growth curve of (**a**) total fermentative yeast for TS1, TS2, TS3 and TS4 (**b**) total fermentative yeast and *Lactobacillus* for sample TS5 (**c**) total fermentative yeast and *Lactobacillus* for sample TS6, (**d**) total fermentative yeast and *Lactobacillus* for sample TS7 and (**e**) total fermentative yeast and *Lactobacillus* for sample TS8 (**f**) total fermentative yeast and *Lactobacillus* for the control sample.

Eight *Tej* samples were produced by inoculating isolated *Lactobacillus* and *Saccharomycetaceae* species in various combinations as a mixed starter culture. Other than pasteurization and inoculation with a defined starter culture, all the processes were conventional. The *Tej* samples from one to four were entirely inoculated by the isolates of *Saccharomycetaceae*. Other samples from five to eight were inoculated by the isolates of both *Lactobacillus* and *Saccharomycetaceae* strains. *Tej* that was spontaneously fermented and widely regarded as the best Ethiopian honey wine was used as a control sample. The details of the production process and the nomenclature of the samples are described in the “Methods” section. Plate count methods for total fermentative yeast and *Lactobacillus* were used to study the interaction of the inoculated microbes during the fermentation period. The total fermentative yeast growth rate for the TS1 sample had the highest exponential growth rate compared to other *Tej* samples inoculated with *Saccharomycetaceae* only (Fig. 3a). Besides, this sample reaches a stationary phase after 14 days of fermentation. Sample TS3 started at a higher cell count of 6 logs CFU/mL and reached the maximum (8 logs CFU/mL) after 14 days of fermentation. Sample TS2 and TS4 showed a gentle growth rate compared to TS1 and TS3. Especially the TS4 sample showed a slower growth rate than other yeast-only inoculated samples. However, this and TS2 samples showed an active growth rate even after 21 days of fermentation (Fig. 3a). Sample TS5, inoculated with a mixed culture of *S. cerevisiae* and *L. hilgardii* strains, grew rapidly in total fermentative yeast and *Lactobacillus* cell count. However, the growth rate of fermentative yeast was by far higher than that of *Lactobacillus*. The cell count of *Lactobacillus* was plateaued at 4 logs CFU/mL after 21 days of fermentation. In contrast, the cell count for total fermentative yeast reaches 7 logs CFU/mL at the end of fermentation (Fig. 3b). A similar higher fermentative yeast count was observed for the TS6 sample compared to its co-inoculated *Lactobacillus* count. After seven days of fermentation, the *Lactobacillus* cell count enters to stationary phase with a cell count of 3 logs CFU/mL. In contrast, the total fermentative yeast of this sample showed a higher growth rate and reached a maximum cell count of 7 logs CFU/mL at the end of its fermentation period (Fig. 3c). A proportional growth rate was observed for yeast and *Lactobacillus* during the TS7 *Tej* sample fermentation period. Nonetheless the growth rate of yeast was higher than the growth rate of *Lactobacillus* (Fig. 3d). At the end of fermentation, this sample recorded 5 and 4 logs CFU/mL count for total fermentative yeast and *Lactobacillus* cell counts, respectively. Sample TS8 started its fermentation with a higher *Lactobacillus* cell count (4 logs CFU/mL) and continued its dominance up to 14 days of fermentation. However, the cell count of fermentative yeast overtook this dominancy after 14 days. At the end of the fermentation period, the total fermentative yeast and *Lactobacillus* count reached 6 and 5 logs CFU/mL, respectively (Fig. 3e). The control sample also showed a similar microbial growth pattern to the previous mixed culture inoculated *Tej* samples. Specifically, the total fermentative yeast was a bit higher (4 logs CFU/mL) than the other inoculated test *Tej* samples. Nevertheless, the final cell concentration for both cell counts was similar to other test samples (Fig. 3f).

### Physicochemical characteristics

The physicochemical properties of *Tej* samples produced by inoculation of different mixed culture combinations are presented in Table 3. Significantly a higher (*P* < 0.05) glucose (14.85 g/L) level was observed for the TS1 sample. In contrast, the TS8 sample (9.85 g/L) and control sample (9.75 g/L) had significantly lower glucose levels (*P* < 0.05). Similarly, the fructose concentration of the TS2 sample was much higher, while the TS8 sample fructose level was significantly lower (*P* < 0.05). Generally, the control and mixed culture fermented samples showed lower sugar content than other inoculated samples (Table 3). Likewise, the ethanol level of the control sample had a significantly higher (*P* < 0.05) ethanol level (10.43 g/100 mL) than other test samples. In contrast, the TS2 sample had a significantly lower (*P* < 0.05) ethanol level of 7.87 g/100 mL than other inoculated samples (Table 3). Compared to samples inoculated with solely yeast culture, samples inoculated with a mixed culture of *Lactobacillus* and *S. cerevisiae* had a greater ethanol content (Table 3). Moreover, the lactic acid levels of fermented honey wines ranged from not detectable to 3.14 g/L. Except for sample TS8 with a very high lactic acid concentration, honey wines inoculated with a mixed culture of *Lactobacillus* and *S. cerevisiae* had roughly the same lactic acid concentration (Table 3). Similarly, the titratable acidity was lower for the samples inoculated with yeast combinations than samples inoculated with *S. cerevisiae* and *Lactobacillus*. Besides, the spontaneously fermented control sample had shown a higher titratable acidity (6.37 g/L).

**Table 3:** The physicochemical properties of fermented honey wine inoculated with various *Saccharomycetaceae* and *Lactobacillaceae* strain combinations.

Parameters	Samples								
	TS1	TS2	TS3	TS4	TS5	TS6	TS7	TS8	Control
Glucose (g/L)	14.85 ± 0.01^a^	13.55 ± 0.01^c^	10.5 ± 0.01^ g^	13.95 ± 0.01^b^	11.75 ± 0.01^f^	12.85 ± 0.01^e^	13.4 ± 0.01^d^	9.85 ± 0.01^ h^	9.75 ± 0.01^ h^
Fructose (g/L)	21 ± 0.01^b^	23.25 ± 0.01^a^	20.1 ± 0 .01^e^	19.95 ± 0.01^f^	20.7 ± 0.01^c^	20.45 ± 0.01^d^	19.7 ± 0.01^ g^	17.35 ± 0.01^i^	19.3 ± 0.01^ h^
Ethanol (g/100 mL)	8.70 ± 0.02^f^	7.87 ± 0.02^i^	8.00 ± 0.03^ g^	9.22 ± 0.02^c^	8.95 ± 0.02^d^	7.96 ± 0.02^ h^	8.81 ± 0.03^e^	9.75 ± 0.03^b^	10.43 ± 0.01^a^
Lactic acid (g/L)	ND	ND	1.09 ± 0.03	0.98 ± 0.01	2.87 ± 0.02	2.67 ± 0.02	2.22 ± 0.01	3.14 ± 0.02	2.93 ± 0.02
pH	3.02 ± 0.01^f^	3.34 ± 0.01^c^	3.66 ± 0.01^a^	3.55 ± 0.01^b^	2.93 ± 0.01^ h^	3.19 ± 0.01^d^	3.00 ± 0.01^ g^	2.84 ± 0.01^i^	3.15 ± 0.01^e^
TA (g/L)	0.96 ± 0.04^ h^	1.14 ± 0.03^ g^	1.31 ± 0.04^f^	0.88 ± 0.04^ h^	4.33 ± 0.05^e^	4.97 ± 0.05^d^	5.10 ± 0.09^c^	6.46 ± 0.04^a^	6.37 ± 0.05^b^

All values are mean ± SD (standard deviation).

Statistical significance difference is compared horizontally.

Significant values are in [Super script Letters].

### Volatile compound and sensory attributes

**Figure 4:**
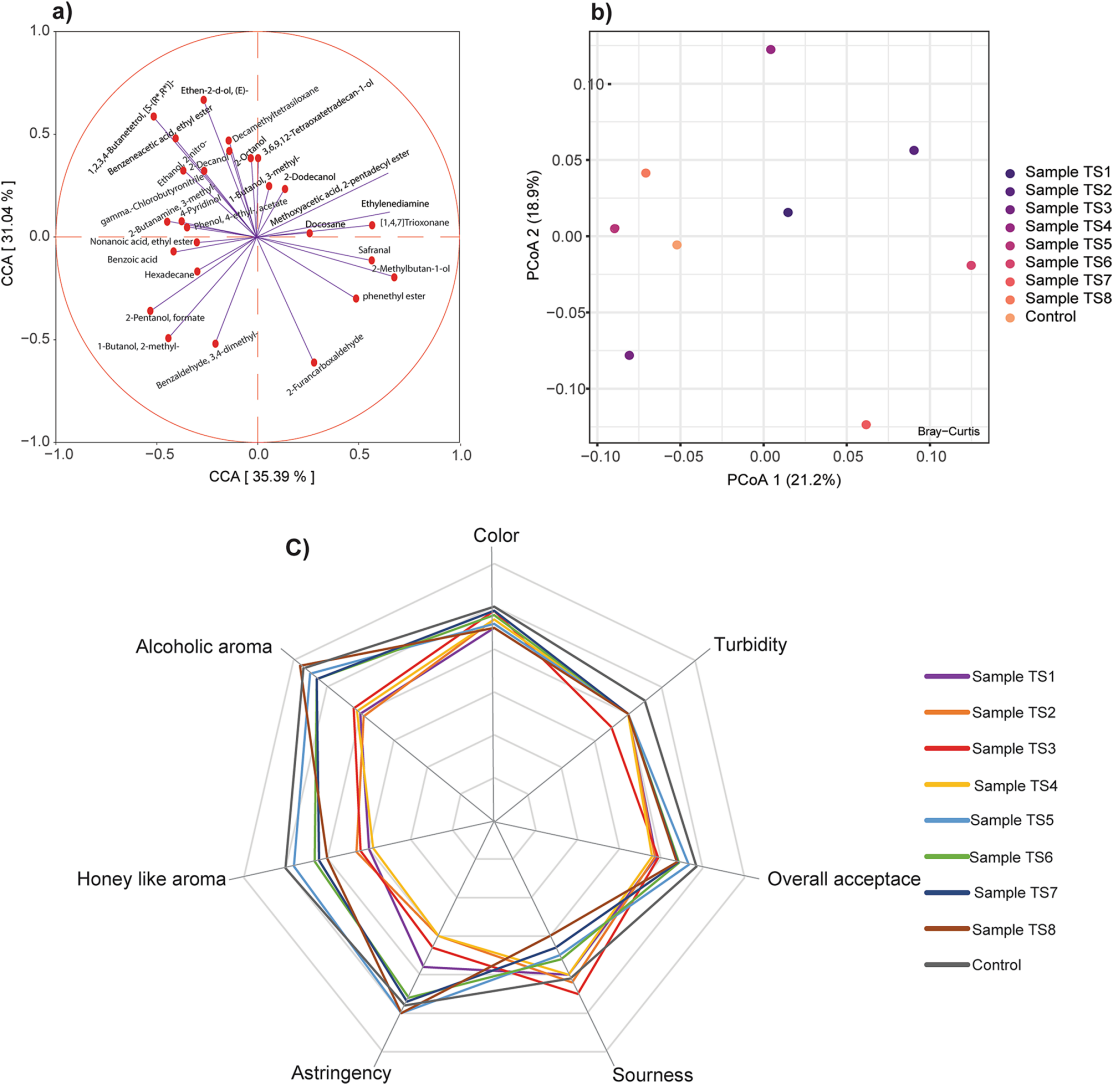
The volatile compound and sensory attributes of honey wine samples inoculated with various strain combinations (**a**) Canonical correspondence analysis (CCA) plot of minor volatile compounds found in a small number of test samples (**b**) Principal coordinate analysis (PCoA) plot with Bray–Curtis dissimilarity for honey wine test samples based on volatile compound concentration (**c**) Radar plot for the sensory analysis results of the test samples using a seven-point hedonic scale.

Esters, alcohols, carbonyl compounds, alkanes, and acids were the major compounds identified and quantified from direct inoculated honey wines samples. From the aforementioned major volatile compounds, alcohols and esters were by far the most dominate volatile compounds in all of honey wines samples (Table 4). For instance, alcohols contributed subtotal values ranging from 728.9 mg/L (control sample) to 577.56 mg/L (TS3 sample). Particularly, ethanol was the most abundant volatile compound identified and quantified in all samples. The mean value of this compound ranged from 321.20 to 448.80 mg/L (Table 4). In this study, phenylethyl alcohol was the second most dominant volatile compound in all fermented honey wine samples. The value of Phenylethyl alcohol ranged from 219.79 to 274.50 mg/L (Table 4). About 13 ester compounds found in all samples, including the control, were identified and quantified. Especially, the ethyl ester of octanoic acid, dodecanoic acid, benzoic acid, and hexanoic acid was the common ester observed for all samples (Table 4). Besides, isoamyl acetate was the major compound for TS1, TS2, TS3, TS6, and control samples. In contrast, this compound was below the detectable limit for the TS7 and TS8 samples. The other volatile compound found in the majority of the samples was alkanes, with average mean values ranging from 13.14 mg/L (TS3 sample) to 43.29 mg/L (TS5 sample). For all fermented honey wine samples, tetradecane was the dominant volatile compound under the alkanes group (Table 4). Although cyclopentasiloxane, dodecamethyl- compound was not detectable in the TS3 sample, it was the most abundant compound in all samples compared to other compounds in the alkanes category (Table 4). Benzaldehyde, which was observed in all samples, was the major volatile compound in the carbonyl group. Furfural, observed in some of the samples, was another minor compound in this category. Volatile acids, which ranged from 16.64 to 29.87 mg/L, were the other significant volatile compounds found in the fermented honey wine sample (Table 4). The volatile phenol 2,4-Di-tert-butyl-phenol, Silanediol, dimethyl-, and Benzene the other significant volatile compound observed almost in all of the samples (Table 4). The volatile compounds that were not observed in the majority of the samples were then plotted on the canonical correspondence analysis (CCA) plot. The compounds such as Benzaldehyde, 3,4-dimethyl-, 1-Butanol, 3-methyl-, 2-Pentanol, Formate, Benzoic acid, Hexadecane were only observed in one or two of fermented honey wine samples (Fig. 4a). Furthermore, a Bray–Curtis principal coordinate analysis (PCoA) plot was utilized to assess dissimilarity between the honey wine tests and control samples based on the detected volatile components. Based on volatile compounds, TS5, TS8, and control samples clustered together on the PCoA plot, especially along the PCoA1 axis (Fig. 4b). Moreover, a subjective analysis of honey wine test samples was performed using a seven-point hedonic sensory score test. Color, turbidity, alcohol aroma, astringency, honey-like aroma, and sourness were the tested sensory attributes of the samples. The color of all test and control *Tej* samples had scored almost a similar result with no significant difference (*P* > 0.05) (Fig. 4c). Nevertheless, there was a significant difference for other sensory attributes between the test samples (*P* < 0.05). Furthermore, TS5, TS6, TS7, and control samples showed a cluster pattern for most sensory attributes (Fig. 4c).

**Table 4:** Volatile compounds found in the majority of honey wine samples inoculated with various *Saccharomycetaceae* and *Lactobacillaceae* strains.

Volatile compounds (mg/L)	Honey wine test samples								
	TS1	TS2	TS3	TS4	TS5	TS6	TS7	TS8	Control
**Esters**									
Hexadecanoic acid, ethyl ester	1.64	2.18	1.82	2.84	2.71	1.99	2.09	2.09	2.27
Acetic acid, 2-phenylethyl ester	1.54	1.42	1.38	1.55	1.72	1.56	1.43	1.45	1.49
Octanoic acid, ethyl ester	13.77	16.99	14.87	15.94	16.40	14.72	15.00	17.01	21.28
Decanoic acid, ethyl ester	0.99	1.23	1.31	1.43	1.32	1.14	1.31	1.24	
Dodecanoic acid, ethyl ester	9.13	12.60	12.34	12.93	13.42	8.20	13.73	11.76	22.63
Methyl salicylate	2.56	2.96	3.29	3.10	3.26	2.55	3.20	3.21	3.58
1-Butanol, 3-methyl-/Isoamyl acetate	29.83	31.07	25.96	3.04	34.14	6.76			35.59
Benzoic acid, ethyl ester	0.85	0.79	0.84	0.86	1.06	0.85	0.91	0.89	0.00
Hexanoic acid, ethyl ester	8.30	7.46	8.16	8.34	10.20	8.28	8.74	8.50	8.78
Tetradecanoic acid, ethyl ester	0.75	0.71		0.85	1.00	0.76	0.87		
Ethyl 9-hexadecenoate	1.01	1.08		1.32	1.33	0.92	1.00		1.03
Ethyl 9-decenoate	1.19	1.36	1.24	1.46	1.45	1.10	1.29	1.34	1.66
Ethyl acetate	0.78	0.61	1.13		1.52	0.68		0.81	0.69
**Alcohol**									
Ethanol	411.20	399.20	321.20	378.40	455.20	430.80	448.40	382.80	440.80
Phenylethyl Alcohol	268.33	244.28	219.79	225.92	270.95	255.00	226.15	243.82	274.50
2-Heptanol	8.72	8.59	9.02	9.18	11.15	8.56	9.42	9.57	
2,3-Butanediol	8.49	9.45	8.97	9.85	11.21	11.00	10.03	12.16	1.22
1-Propanol, 2-methyl-	11.78	11.39	11.92	11.97	14.21	12.00		12.47	12.38
**Carbonyl compounds**									
Benzaldehyde	20.16	21.90	21.45	22.42	24.31	18.98	19.39	19.76	19.06
Anisaldehyde	7.95	7.51			9.02	8.80	9.09	9.07	8.75
Acetoin	0.76	0.71	1.17	0.82		0.72			
Furfural	0.72		0.98			0.74		0.77	0.75
**Alkanes**									
Cyclohexasiloxane, dodecamethyl-	15.62	17.32		16.19	14.10	14.12	15.04	16.63	15.55
Cyclooctasiloxane, hexadecamethyl-	1.12	1.06	0.94	0.82	1.01		1.33	0.82	0.73
Cyclopentasiloxane, decamethyl-	0.98	0.97	2.12	0.97	0.94	0.93		0.97	0.92
Tetradecane	9.20	7.79	10.09	7.89	15.26	13.38	13.79	10.64	9.23
Hexadecane	0.79	0.66		0.75	0.00	0.87	0.87	0.00	0.74
Dodecane	7.60	6.88		7.26	11.99	9.88	11.13	8.98	8.43
**Other Compounds**									
2,4-Di-tert-butylphenol	38.82	42.09	28.34	41.22	44.15	36.34	33.50	38.21	38.35
15-Crown-5	1.04	0.62		1.22	0.77		0.86		
12-Crown-4	0.75	0.72	0.91	0.69			0.83	0.73	0.69
Silanediol, dimethyl-	11.94	10.69	16.43	12.18	13.21	10.21	8.96	13.36	13.26
Benzene	11.02	11.56	13.44	12.37	14.46	12.02	7.68	12.36	12.50
α-Terpineol	7.04	6.17			6.98		7.69	6.94	7.20

All values are the means of the duplicates.

Table spaces left open is for the values below the detectable limit.

## Discussion

Ethiopian honey wine, *Tej*, is produced by a spontaneous fermentation process^4,5^. However, this kind of spontaneous fermentation has the main drawback on the predictability and controllability of the process^12^. To shift from spontaneous to direct fermentation, without losing any of its major quality attributes, studying the microbial ecology and development of starter culture is the major task^1^. The previous studies on *Tej* samples had clearly pointed out the microbial ecology was dominated by the species of *Saccharomyces* and *Lactobacillus*^7,9,15^. Thus, developing a starter culture composed of a mixture of *Lactobacillus* and *Saccharomyces* species will help achieve a direct *Tej* fermentation system without scarifying the wholesomeness of the final product.

The strains of *Saccharomycetaceae* (*S. cerevisiae*, *W. anomalus,* and *P. fermentans*) were isolated from *Tej* samples. These isolates were obtained after subjecting many isolates to intensive screening. Since they are the most responsible species for converting carbohydrates to ethanol, *S. cerevisiae* is the most expected strain in every alcoholic beverage^16^. Similar *S. cerevisiae* isolates were obtained from *Tej* and other Ethiopian traditional beverages^4,17^. Although it is uncommon to use *W. anomalus* and *P. fermentans* isolates as the sole starter culture for alcoholic beverage fermentation, co-culturing them with *S. cerevisiae* to improve the flavor of the alcoholic beverage is currently attracting the interest of many scientists^18–20^. Thus, the isolated *Saccharomycetaceae* strains have a good potential to be used as a starter culture for the fermentation of Ethiopian honey. However, *W. anomalus and P. fermentan*s were not isolated from all sample collection areas. This could be due to the inherent microbial variability of the samples caused by the spontaneous nature of fermentation, or it could also be due to the stringent screening procedure^4^.

*Lactobacillus* species (*L. hilgardii*, *L. paracasei,* and *L. parabuchneri*) were the other strains isolated from *Tej* samples. Usually, lactic acid bacteria are introduced to wine after the completion of alcoholic fermentation or at the start of malolactic fermentation^21^. However, in Ethiopian honey wine, *Lactobacillus* and *Saccharomyces* are involved in the fermentation from the beginning up to the end of the fermentation period^9^. Thus, isolating *Lactobacillus* strains from *Tej* samples was not a surprising result. Especially, strains of *L. hilgardii* had been commonly involved in the alcoholic fermentations^22^. Besides, all of the isolates were under the heterofermentative *Lactobacillus* category. The *Lactobacillus* under this category has the ability to produce lactate, ethanol, and carbon dioxide from a given carbon source^23^. Furthermore, some of the *Lactobacillus* species enhance the flavor of the produced alcoholic beverage^24^. Similar lactic acid bacterial isolates for the *Tej* sample were obtained by applying phenotypic microbial characterization techniques^4^.

The *Lactobacillus* isolates clonal relationship were studied by using RAPD-PCR DNA fingerprinting. Because it amplifies fragments of genomic DNA, RAPD is one of the most powerful tools for studying genetic variation in living organisms^25^. However, there is still a loophole of this method on the reproducibility due to mismatch in annealing^26^. In this study, the isolated *Lactobacillus* strains of the same species did not show a high level of polymorphism. The primary justification that could be forwarded for this result could be due to intensive screening for harsh environment might cause isolated strains to have a similar allelic diversity. The luck of reproducibility, accuracy and application of limited number of primers during RAPD-PCR amplification could also be the other factor for this particular result^25,26^. The genetic diversity of *S. cerevisiae* strains was also studied by using Microsatellite multiplex PCR. Microsatellites, also known as simple sequence repeats (SSRs), are short repeats of DNA sequence motifs found in many genomes^27^. Among six *S. cerevisiae* strains three of them had shown high level of polymorphism. The inherent high genetic diversity of *S. cerevisiae* strains could be the possible major reason for these variations. However, the number of analyzed loci still had the influence on the genetic diversity of the strains^28^. Thus, it is worth to know that, the current result could have been different if more than three loci of the total genome had been amplified. One of the three *S. cerevisiae* strains (strain code AAF18) that showed similar allelic diversity was chosen for inoculation and phenotypic characterization in this study.

In addition to the aforementioned biochemical characterization of isolates, further characterization was performed using phenotypic microarray. The metabolism of *S. cerevisiae* and *P. fermentans* was high in the presence of vitamins (D-biotin, nicotinamide, thiamine), amides ((5)4-amino imidazole-4(5)-carboxamide), and phosphorous compound (pyrophosphate). The high metabolism could be due to the micronutrient (vitamins and minerals) requirements of microbe for the proper metabolism of cells^29^. Moreover*, Saccharomycetaceae* and *Lactobacillus* isolates had exhibited an excellent osmotic tolerance ability. This tolerance could be due to the adaptation of isolates to higher osmotic pressure experienced during honey wine fermentation. However, a lower magnitude of metabolism for all isolates was observed for the growth medium containing greater than 6% NaCl concentration. This result could be due to the cellular metabolic shift of isolated microbes from hyperplasia to hypertrophy at higher NaCl concentration^30^. Since honey-must has a lower pH by itself, and the pH continues to drop as fermentation progresses, tolerance to acidic stress is one of the essential characteristics of isolates to look for when choosing a starter culture. However, a lower pH can charge the biological molecules, which later can negatively impact both structure and function of cells^31^. In this study, *Saccharomycetaceae* responded well to lower pH environments than *Lactobacillus* isolates. This response could be due to the better membrane stability of fungi over bacteria. Especially for a pH lower than 4.5, the magnitude of metabolism for *Lactobacillus* isolates was very low. *Pichia fermentans,* conversely, had a strong tolerance for low pH than any other isolates. A similar *Pichia* isolates tolerance over the other *Saccharomyces* isolates was observed in the previous study^32^.

Following the extensive characterization of isolated microorganisms, it had been discovered that they had a strong chance of being employed as a starter culture for *Tej* fermentation. However, their growth in honey-based media and interaction with each other was the important concept to be covered for developing a starter culture. Fermentative yeast and *Lactobacillus* count were performed five times throughout the fermentation period of *Tej*. The availability of ample nutrients and little microbial competition causes a higher fermentative yeast count for the TS1 sample, especially at the early fermentation stage^33^. A similar increment of fermentative yeast for sample TS3 could be due to a higher initial microbial count of inoculum and higher availability of the required nutrient^33,34^. Surprisingly, the slower growth rate of fermentative yeast for sample TS4 could be caused by either mutual inhibition or the slow growth rate of one of the inoculums. The fast growth rate of *Saccharomyces* was observed compared to that of *Lactobacillus* in a mixed culture inoculated *Tej* fermentation. This fast growth rate could be caused either by the inhibition of *Lactobacillus* by *Saccharomyces* species or due to the inherent fast growth rate characteristics of *Saccharomyces*^35,36^. However, the growth rate of *Lactobacillus* itself followed almost a similar pattern as it was discussed in the previous study on *Tej* microbial dynamics^9^. Similar studies on the co-inoculation of *Saccharomyces* and *Lactobacillus* had revealed a production of good quality characteristics alcoholic beverage^37^. Moreover, the final product physicochemical and volatile compound analysis of *Tej* samples was performed to assess whether or not the inoculated fermentation had met the intended goal. In terms of residual sugar concentration, all test samples fell within the range observed in previous survey studies for *Tej* samples^6–9^. This result is essentially a positive testimony for the ability of the inoculum to utilize carbon sources from honey for cellular growth and the production of exo-metabolic products^38^. Similarly, test samples showed good ethanol concentrations, particularly for the product inoculated with a mixed culture of *S. cerevisiae* and *Lactobacillus* isolates; this could be due to the co-inoculation of heterofermentative *Lactobacillus* isolates, resulting in additional ethanol production^39^. Besides, the substantial lactic acid concentration was especially observed for the samples co-inoculated with *S. cerevisiae* and *Lactobacillus* isolates and the control sample. Moreover, TS3 and TS4 samples also showed the same lactic acid concentration, probably due to the lactic acid-producing yeast or due to lactic acid bacteria trace contamination. Similar lactic acid production by yeast was observed during the fermentation of beer and mead^40,41^.

The volatile compound analysis will help to objectively investigate the influence of different inoculum combinations on fermented *Tej* samples. The volatile compound of the honey wine is derived either from raw materials or from fermentative microorganisms^42^. Nevertheless, esters and alcohols were the most dominant volatile compounds in all test samples, including those of the test and control samples. Since honey wine is an alcoholic beverage, the presence of alcohol and alcohol derivative volatile compounds is expected to observe^42,43^. During alcoholic fermentation and slow aging, alcohol acetyltransferase and other enzymes could catalyze the formation of esters from an activated fatty-acyl CoA compound and alcohol^44^. Isoamyl acetate, the most abundant ester in *Tej* samples, is the compound responsible for fruity and floral notes and is typically derived from yeasts^45^. Moreover, Octanoic acid, ethyl ester, the second most abundant ester found in all samples, give pear, brandy, and lentil flavor to the produced *Tej*^46^. This volatile compound is usually the product of the fermentation process^47^. Esters, which contribute to the fruity aroma of wines, are generally good indicators of fermented beverages' young age^48^. Like honey wine samples treated with different fining agents^49^, alcohol was also the most dominant volatile compound in all of *Tej* samples. The dominant alcohols (ethanol and phenylethyl alcohol) were the compounds responsible for the vinous odor and pungent taste of alcoholic beverages^50^. Furthermore, higher alcohols (1-Propanol, 2-methyl-, 2,3-Butanediol, and 2-Heptanol) in the tested samples also contributes to the good flavor of honey wine^49^. These higher alcohol content and proportion played a significant role in improving the wine taste^51^. Besides esters and ethanol, other volatile compounds including carbonyl groups independently and interactively play a none dimensioning effect for the test and flavor of honey wine^52^. Furthermore, alkanes were also the other major group of volatile compounds observed in *Tej* samples. Cyclopentasiloxane compounds, in particular, were found in almost all of the samples. These silicon-based alkenes in the samples could be due to GC column leaching or bleeding. However, other studies on the volatile compounds of fermented beverages products have also found these cyclopentasiloxane base compounds in their test samples^52,53^. The volatile compound absent in the majority of the samples and with a lower concentration was plotted on the CCA plot. The majority of these compounds (3,4-dimethyl-, 1-Butanol, 3-methyl-, 2-Pentanol, Formate, Benzoic acid) plotted on CCA are volatile compounds derived from microbial fermentation process^54^. Furthermore, TS5, TS8, and control samples showed some kind of cluster on the Bray–Curtis dissimilarity PCoA plot. The clustered sample is a good indication of the similarity of the compounds for the samples mentioned above because the PCoA plot was generated from total volatile compounds^55^. The sensory analysis also revealed that these samples had a cluster pattern on the radar plot of a seven-point hedonic scale score for alcoholic aroma, astringency, sourness, and overall acceptance of sensory quality attributes. These sensory attributes are actually the results of volatile compounds present in the samples^56^. Specifically, these samples were made from honey-must fermentation inoculated with a mixed culture (*Saccharomyces* and *Lactobacillus*).

## Conclusions

The isolated strains of *Saccharomycetaceae* and *Lactobacillaceae* for the purpose of starting honey wine fermentation had demonstrated good tolerance to osmotic pressure and a lower pH environment, as well as requiring minimal micro-nutrition These isolates, with different combinations, were applied to honey-must to assess its ability to produce a good quality *Tej*. Generally, test samples inoculated with a mixed culture of *Saccharomyces* and *Lactobacillus* strains had lower residual sugar content and higher ethanol level. Besides, the volatile compounds and sensory attributes for these samples were consistent with the control sample. Specifically, TS5 and TS8 samples, inoculated by different combinations of *S. cerevisiae*, *L. hilgardii*, *L. parabuchneri,* and *L. paracasei*, had a close quality attribute resemblance with that of the control sample. Thus, Ethiopian honey wine can be fermented using a direct inoculation system with either *S. cerevisiae* and *L. hilgardii* or *S. cerevisiae* and other *Lactobacillus* isolates (*L. hilgardii*, *L. parabuchneri*, and *L. paracasei*) without losing its major quality attributes.

## Methods

### Sample collection, transportation and storage

A total of 21 *Tej* samples containing presumptive starter culture strains were collected aseptically from three locations in Ethiopia (Addis Ababa, Bahir Dar and Debre Markos) using sterile screwed cap. These samples were obtained from alcohol retailers willing to sell their product for research purposes. All the *Tej* samples were then transported to the point of analysis via ice box containing freeze pack. Besides, samples which needs further analysis were stored in a refrigerator at temperature of 4 °C.

### Enumeration and isolation of presumptive starter cultures

Yeast enumeration and isolation were carried out using YPD agar^57^. Chloramphenicol (100 μg/mL) was added to the growth media after autoclaving and cooling to 50 °C to inhibit bacterial growth. Each *Tej* sample (1 mL) was mixed with 9 mL of sterile saline solution (0.85% NaCl) to produce a serially diluted inoculating sample. After inoculation, the pet reddish was incubated at a temperature of 30 °C for a period of 48 h. At the same time, presumptive *Lactobacillus* enumeration and isolations were done via MRS agar medium and incubated under the anaerobic condition at 30 °C for 72 h. The macroscopic features (shape, size, pigment, surface, elevation, and opacity) of yeast and *Lactobacillus* colonies of different types were streaked on the same respective mediums. About 15 colonies of each representative type were re-streaked on the same medium to obtain pure colonies.

### Phenotypic and physiological characterization

Presumptive *Lactobacillus* isolates were subjected to gram reaction, catalase activity, and indole tests according to methods described by Saarisalo et al. (2007)^58^. Then gram positive, catalase, and indole negative bacterial together with yeast isolates were subjected to purpose-oriented screening. The ability of isolates to ferment carbohydrate and to produce carbon dioxide (CO_2_) was conducted using Durham tubes according to standard protocol^59^. Then, both isolates (presumed yeast and *Lactobacillus*) with the ability to ferment glucose, and sucrose and produce CO_2_ were passed on to the next stage of the screening process. The ability to grow at high sugar concentrations was tested by applying 10%, 18%, and 25% glucose on growth media^60^. The microbial isolates with high sugar concentration tolerance were subjected to a third test, which was the ability to grow at lower pH levels of 3.5, 4.0, and 4.5. Both bacteria and yeast isolated with the ability to grow at a lower pH were subjected to a temperature sensitivity test. Thus, all isolates screened using the methods mentioned above were further tested for their ability to grow at temperatures of 15 °C, 25 °C, and 35 °C. Isolates that performed well in the above purpose-oriented screening were further subjected to genotypic characterization.

### Genotypic identification and characterization

After extracting DNA from the isolated strains, ITS and 16sRNA amplicon sequencing were performed for the identification of yeast and *Lactobacillus* isolates, respectively. The identified *Lactobacillus* strains were then subjected to RAPD-PCR analysis by applying the methods used by Kostinek et al.^61^. Similarly, all *S. cerevisiae* isolates were subjected to microsatellite multiplex PCR analysis using the method developed by Vaudano and Garcia-Moruno^62^. Three microsatellite loci, SCPTSY7, SC8132X, and YOR267C were used for their high degree of polymorphism. Both RAPD-PCR and microsatellite multiplex PCR products were separated by using 1.8% (w/v) agarose gel electrophoresis system. After pattern processing and cluster analysis, a dendrogram was developed by using UPGMA method.

### Phenotypic microarray analysis

PM assay for our isolates was performed by following manufacturer protocol of PM Technology (Biolog, Hayward, CA, USA). PM micro-panels are 96-well microplates with different substrates in each well. This study used PM5, PM9, and PM10 assays to determine nutrient requirements, osmotic/ionic stress responses, and sensitivity to different pH environments. Each well of the panels contains the required minimal medium components, specific dye, and unique substrate. The DNA amplicon sequenced bacterial and yeast isolates from *Tej* samples were chosen for this analysis. All test strains were first incubated overnight at a temperature of 30 °C on SMA (Standard Methods Agar, BioMérieux) medium. The cells were then collected from the SMA medium with a sterile cotton swab and dispersed in a sterile capped tube containing 20 mL of the inoculation fluid (IF-0, Biolog Inc.). Using a Biolog turbidimeter, the cell concentration was adjusted to 81% transmittance after all the bubbles created during cell dispersion had settled. After that, PM5, PM9, and PM10 plates were inoculated with the cell suspension (100 μL per well) and incubated at a temperature of 33 °C for about 120 h in the Omnilog Incubator/Reader (Biolog Inc., Hayward, USA). The color changes in the wells were measured once every 15 min, allowing for both amplification and quantification of the phenotype using OmniLog® data collection Software v 1.2 (Biolog Inc.).

### Mixed culture fermentation

#### Starter culture preparations

Three *Lactobacillus* isolates were first inoculated into a 250 mL conical flask with MRS broth and incubated at 30 °C for 24 h under anaerobic conditions. Similarly, three yeast isolates were inoculated into a 250 ml Erlenmeyer flask with YPD broth and incubated at a temperature of 30 °C for 18 h. Following the incubation period, the growing mediums were centrifuged at 3500 rpm for 10 min to obtain a higher cell concentration. It was then washed twice using sterile water and resuspended again in saline solution (0.85% NaCl). Finally, the concentration of the suspended inoculums was adjusted to achieve the required final microbial concentrations.

#### Inoculated *Tej* fermentation

*Tej* was made in the lab by using inoculated fermentation and the same procedures as when it was made traditionally^3,5,7,9^. The same honey-must was used for all of the samples, which was made by mixing honey and water in a 1:3 ratio, and the concentrations of glucose and fructose were obtained 155 g/L and 210 g/L, respectively. Then, this mixture was pasteurized at 65 °C for 10 min. The pasteurized honey-water mixture was then cooled and aseptically transferred to a ten-piece set of 50-mL conical flasks with top screw caps. Taking the previous literatures^7–9^ and preliminary findings as a baseline, the prepared inoculums were mixed with different strain combinations at varying concentrations to create eight *Tej* samples. The first *Tej* sample (TS1) was prepared by inoculating *S. cerevisiae* (3 log cfu/mL) to honey-must mixture. The second sample (TS2) was inoculated with *S. cerevisiae* (3 log cfu/mL) and *P. fermentans* (2 log cfu/mL). The third sample (TS3) was inoculated with *S. cerevisiae* (3 log cfu/mL), *P. fermentans* (1 log cfu/mL), and *W. anomalus* (2 log cfu/mL). The fourth sample (TS4) was inoculated with *S. cerevisiae* (3 log cfu/mL) and *W. anomalus* (2.5 log cfu/mL). The fifth sample (TS5) was inoculated by *S. cerevisiae* (3 log cfu/mL) and *L. hilgardii* (2 log cfu/mL). The sixth test sample (TS6) was inoculated with *S. cerevisiae* (3 log cfu/mL) and *L. parabuchneri* (2 log cfu/mL). Similarly, sample seven (TS7) was inoculated with *S. cerevisiae* (3 log cfu/mL) and *L. paracasei* (2 log cfu/mL). The last experimental *Tej* sample (TS8) was prepared by inoculating a honey-water mixture with *S. cerevisiae* (3 log cfu/mL), *L. hilgardii* (2 log cfu/mL), *L. parabuchneri* (1 log cfu/mL)and *L. paracasei* (1 log cfu/mL). After inoculation, they were allowed to finish their primary fermentation stage for about 4 days at room temperature (26 °C). At this time, 1.5 g of boiled and sterilized gesho leaves and stems were added to the fermentation mediums (50 mL). The *Tej*-making process was completed after allowing these mixtures to spend for 20 more days.

### Physicochemical analysis

#### Sugar analysis

The concentrations of glucose and fructose in the samples were determined using a High-Performance Liquid Chromatography-Evaporative Light Scattering Detector (HPLC-ELSD) system^63^. After centrifuging 5 mL *Tej* sample at 3200 rpm for 30 min, the supernatant was double filtered through 0.45 µm and 0.22 µm pore size membranes. Analyses were performed using an LC-20AT HPLC (Shimadzu, Japan), a YMC-Pack Polyamine II (250 4.6 mm. D.S-5 m, 12 nm) column, and an ELSD-LT II detector. At a column temperature of 30 °C, a 10 μL aliquot of the samples was injected into the HPLC-ELSD system. A mobile phase of acetonitrile: water at a 75:25 ratio with an isocratic pressure and a flow rate of 1.0 mL/min was used during the analysis period. Nitrogen (N_2_) was used as the nebulizer gas with a flow rate of 2 L/min. Finally, sugar quantification was performed by comparing the sugar peak areas of the samples to the corresponding standards calibration curves.

#### Ethanol quantification

The ethanol level was determined using the standard method developed by OIV (2020)^64^. A volumetric flask was first filled with a 200 mL aliquot of the *Tej* sample. The measuring cylinder was then rinsed three times with 20-mL distilled water. Additional distilled water (20 mL) was added to the distillate collection volumetric flask (200 mL). This collection flask was immersed in a cold-water bath throughout the distillation process. The distillate collection volumetric flask was then filled with deionized water to the markup. Finally, the alcohol content was determined using a standard aqueous organic solution table.

#### Lactic acid quantification

The lactic acid concentration in the *Tej* samples were quantified using spectrophotometric method developed by Borshchevskaya et al.^65^. A calibration curve was first constructed using serially diluted known concentration of DL-lactic acid stock solution (89%, ρ = 1.2 g/mL, Sigma-Aldrich). The absorbance of each serially diluted lactic acid stock (50 µL) solution was measured at 390 nm after it was mixed with 2 mL of a 0.2% solution of iron (III) chloride (FeCl_3_.6H_2_O). Similarly, the *Tej* samples were first centrifuged to remove the cellular particles from the fermented aliquot. The separated supernatant was then diluted 20-fold using deionized water. Subsequently, the test samples (50 µL) were mixed with 2 mL of a 0.2% solution of iron (III) chloride, and the absorbance at 390 nm was measured compared to the reference solution (2 mL of a 0.2% solution of FeCl_3_·6H_2_O). The reaction and measurements were conducted at a temperature of 25 °C ± 5 °C. Finally, the lactic acid concentration was calculated using a calibration curve that accounted for the 20-fold dilution of the *Tej* sample.

#### Volatile aromatic compound profiling

Volatile aromatic compounds were profiled using a headspace solid-phase microextraction coupled with gas chromatography-mass spectrometry (HS–SPME–GC–MS). *Tej* samples produced using various inoculum combinations were examined for volatile aromatic compounds by adapting the methods used by Ravasio et al.^66^. Briefly, 2.5 mL of the *Tej* sample and internal standard of 2-octanol (10 µL) was placed in a 20 mL head space extraction glass vials. Samples were then equilibrated for 10 min at 40 °C and 250 rpm. The extract was then adsorbed for 30 min at 40 °C using 50 µm layer DVB/CAR/PDMS fiber. After extraction, the fibers were drawn into the needle and transferred to the injection of the GC–MS system for desorption at 250 °C for 5 min. Chromatographic separation was performed with a TRACE TR-5 GC column (30 m,0.25 mm × 0.25 μm), using helium as carrier gas at 1.0 mL/min. The oven temperature program was set as follows: 50 °C to 220 °C, at 2 °C/min, then raised to 240 °C, at 10 °C /min and held for 10 min. The total run time was 91 min. The mass spectrometer (quadrupole) operated in full scan mode, detecting fragments in a mass range of 35 to 500 m/z. The Internal standard was used to semi-quantify volatile aromatic compounds following the method used by Pino and Barzola-Miranda^67^. The final identification of aromatic compounds was conducted by comparing mass spectra with the NIST 14 library and chemical standards.

### Sensory analysis

The acceptability of *Tej* samples fermented by direct inoculation of isolated strains was assessed using consumer-based sensory analysis. Twenty-four experienced consumers were chosen to evaluate the sensory attributes of the test samples. The evaluations were conducted in a sensory panel room at 25 °C. About 10 mL *Tej* samples were served to the panelist using transparent plastic cup. The samples were evaluated twice after being blind-coded with three-digit random numbers. The panelists were then asked to rate each sample, with a seven-point hedonistic scale, for the (a) color (b) turbidity (c) alcoholic aroma (d) honey like aroma (e) astergency and (f) overall acceptance.

### Statistical analysis

Except for the volatile compound analysis, which was done in duplicate, all other experimental analyses were done in triplicate. The collected data were then checked for normality and homoscedasticity (Levene’s test)^68^. Subsequently, means of the samples data were compared by Duncan’s multiple comparison test. The statistical significance (*P* < 0.05) was later determined by a one-way analysis of variance (ANOVA). The data analysis, CCA and PCoA plots were performed using RStudio 4.0.3 software.

## Data Availability

The datasets generated and/or analyzed during the current study are available in the National Center for Biotechnology Information (NCBI) repository with Accession Number PRJNA858936, and PRJNA858953.
